# PlantCAZyme: a database for plant carbohydrate-active enzymes

**DOI:** 10.1093/database/bau079

**Published:** 2014-08-14

**Authors:** Alexander Ekstrom, Rahil Taujale, Nathan McGinn, Yanbin Yin

**Affiliations:** ^1^Department of Computer Science and ^2^Department of Biological Sciences, Northern Illinois University, DeKalb, IL 60115, USA

## Abstract

PlantCAZyme is a database built upon dbCAN (database for automated carbohydrate active enzyme annotation), aiming to provide pre-computed sequence and annotation data of carbohydrate active enzymes (CAZymes) to plant carbohydrate and bioenergy research communities. The current version contains data of 43 790 CAZymes of 159 protein families from 35 plants (including angiosperms, gymnosperms, lycophyte and bryophyte mosses) and chlorophyte algae with fully sequenced genomes. Useful features of the database include: (i) a BLAST server and a HMMER server that allow users to search against our pre-computed sequence data for annotation purpose, (ii) a download page to allow batch downloading data of a specific CAZyme family or species and (iii) protein browse pages to provide an easy access to the most comprehensive sequence and annotation data.

**Database URL**: http://cys.bios.niu.edu/plantcazyme/

## Introduction

Lignocellulosic biofuels have received great attentions in the past decade for obvious economic and environmental reasons [1]. Other than using starch-based plant materials as the feedstock, lignocellulosic biofuels use inedible plant biomass materials, which however are very recalcitrant to be degraded to release fermentable sugars. The bioenergy research community thus has major interests in genetically modifying plants in order to develop low-cost biofuels [2]. To achieve this goal, researchers need to know which genes should be modified to acquire the desired plants with lower recalcitrance to enzymatic degradation. Therefore biomass-related enzyme databases are highly needed to promote the development of transgenic biofuel crops [3]. Carbohydrate-Active enzymes (CAZymes) are enzymes responsible for the synthesis, degradation and modification of storage and structural biomass polysaccharides [4] and thus are the most important enzymes for bioenergy research. CAZymes are not only found in plants and bacteria, but also in fungi and animals, responsible for the synthesis, degradation and modification of all the glycoconjugates in nature including glycoproteins and glycolipids. Therefore they are also fundamentally important for general carbohydrate and glycobiology research [4].

CAZymes are present in all life kingdoms and particularly abundant in plants [5]. Since 1998, the CAZyme database, known as CAZy, has started to collect experimentally (biochemically, genetically and structurally) characterized CAZyme proteins and classify them into protein families and so far has created 330 families (as of May 2013) of six classes based on sequence homology: GHs (glycoside hydrolases), GTs (glycosyltransferases), CEs (carbohydrate esterases), PLs (polysaccharide lyases), AAs (auxiliary activities) and CBMs (carbohydrate binding modules) [6]. It then populated each family by including homologs from GenBank, UniProt and PDB databases using both BLAST and protein domain/motif search strategies as well as expert manual inspection of sequence alignment [4, 7]. CAZy is an extremely useful resource for its most original classification scheme and high-quality manual curation, and thus has been widely accepted by the carbohydrate research community.

A great demand of an automated CAZyme annotation emerged in the past few years due to the production of thousands of completed plant and microbial genomes and metagenomes. However CAZy database does not provide automated CAZyme annotation. In view of this need, in 2012 we have developed a web server named dbCAN, to allow users to submit the newly sequenced genomes for an automated CAZyme annotation [8]. Behind the web server are hidden Markov models (HMMs) of the 330 CAZyme families; each HMM represents the sequence alignment of conserved signature domains of each family, which were retrieved from annotated CAZyme protein sequences of the CAZy database. dbCAN website has received over thousands of visits from many countries after publication, demonstrating its impact on the research of CAZymes.

The availability of the 330 CAZyme HMMs has also made it possible to build a dedicated database for plant CAZymes. With regard to similar resources, the CAZy database covers only two (*Arabidopsis thaliana* and *Oryza* *sativa*) out of over 40 sequenced plant and algal genomes; all sequenced bioenergy crops (e.g. poplar, switchgrass, soghum) and evolutionarily important organisms (e.g. moss, spike moss, algae) were not included. Two other databases, pDAWG [9] and Rice GT [10], are limited to a small number of CAZyme families and genomes. There are also a few other databases such as the Cell Wall Genomics database [11] and the Cell Wall Navigator database [12], which only contain a very small number of CAZyme families. Therefore, the development of PlantCAZyme is a timely and highly significant addition to the toolbox of plant carbohydrate and bioenergy research.

## Construction and Content

### Collection of CAZyme sequences

Over 40 plant and algal genomes are completed and most of them are available in the Phytozome database [13]. To collect the plant CAZyme protein sequences, we used 330 dbCAN HMMs as query and scanned 35 genomes (Table 1), including 34 Phytozome genomes of 23 dicots, six monocots, one moss, one spike moss, two chlorophyte algae, as well as one gymnosperm genome [14] that is not available in Phytozome, using the HMMER 3.0 package as the homology search tool [15] with default parameters (*E*-value < 10 and output in parseable table of per-domain hits). The HMMER output was further processed to keep the significant hits as described in below.

**Table 1.** Thirty-five plant and algal genomes that are included in the PlantCAZyme database

*Species*	Clade	Source	# of genes	# of CAZyme genes	% of CAZyme genes
*Volvox carteri*	Chlorophyte	Phytozome	14 971	198	1.32
*Chlamydomonas reinhardtii*	Chlorophyte	Phytozome	20 497	285	1.39
*Physcomitrella patens*	Bryophyta	Phytozome	21 173	857	4.05
*Selaginella moellendorffii*	Lycophyta	Phytozome	22 285	919	4.12
*Picea abies*	Gymnosperm	Congenie	71 158	1843	2.59
*Aquilegia coerulea*	Dicot	Phytozome	24 823	1099	4.43
*Arabidopsis lyrata*	Dicot	Phytozome	32 670	1232	3.77
*Arabidopsis thaliana*	Dicot	Phytozome	27 416	1224	4.46
*Brassica rapa*	Dicot	Phytozome	40 905	1812	4.43
*Capsella rubella*	Dicot	Phytozome	26 521	1211	4.57
*Carica papaya*	Dicot	Phytozome	27 769	845	3.04
*Citrus clementina*	Dicot	Phytozome	24 553	1098	4.47
*Citrus sinensis*	Dicot	Phytozome	25 379	1083	4.27
*Cucumis sativus*	Dicot	Phytozome	21 503	1008	4.69
*Eucalyptus grandis*	Dicot	Phytozome	36 376	1711	4.70
*Fragaria vesca*	Dicot	Phytozome	65 662	1105	1.68
*Glycine max*	Dicot	Phytozome	54 175	2354	4.35
*Gossypium raimondii*	Dicot	Phytozome	37 505	1648	4.39
*Linum usitatissimum*	Dicot	Phytozome	43 471	2018	4.64
*Malus domestica*	Dicot	Phytozome	63 514	2220	3.50
*Manihot esculenta*	Dicot	Phytozome	30 666	1442	4.70
*Medicago truncatula*	Dicot	Phytozome	44 135	1173	2.66
*Mimulus guttatus*	Dicot	Phytozome	26 718	1271	4.76
*Phaseolus vulgaris*	Dicot	Phytozome	27 197	1351	4.97
*Populus trichocarpa*	Dicot	Phytozome	41 335	1751	4.24
*Prunus persica*	Dicot	Phytozome	27 864	1288	4.62
*Ricinus communis*	Dicot	Phytozome	31 221	1135	3.64
*Thellungiella halophila*	Dicot	Phytozome	26 351	1132	4.30
*Vitis vinifera*	Dicot	Phytozome	26 346	1096	4.16
*Brachypodium distachyon*	Monocot	Phytozome	26 552	1243	4.68
*Oryza sativa*	Monocot	Phytozome	39 234	1363	3.47
*Panicum virgatum*	Monocot	Phytozome	65 878	2624	3.98
*Setaria italica*	Monocot	Phytozome	35 471	1487	4.19
*Sorghum bicolor*	Monocot	Phytozome	27 608	1334	4.83
*Zea mays*	Monocot	Phytozome	39 656	1475	3.72

### Selection of golden standard datasets for accuracy benchmark

Since the CAZymes of *Arabidopsis* and rice have been annotated in the CAZy database, we have used these two genomes to calculate the sensitivity (or recall) and positive predictive value (or precision) of our CAZyme data. It is worth mentioning that the ‘annotated’ CAZymes of CAZy include not only experimentally characterized proteins, but also proteins that are deemed to be true homologs of the characterized proteins. For example, there are only three *Arabidopsis* proteins experimentally characterized to be GH17 enzymes (http://www.cazy.org/GH17_characterized.html); however 51 *Arabidopsis* proteins are listed as GH17 enzymes (http://www.cazy.org/GH17_eukaryota.html). The reason is that CAZy database annotates CAZymes from the GenBank database, including those from *Arabidopsis* and rice, by combining homology search and expert curation (e.g. manual inspection of sequence alignment for characteristic amino acid motifs [7]). Most of the *Arabidopsis* CAZymes including those experimentally uncharacterized have been manually curated by CAZy developers and published in 2001 [16]. The similar approach has also been applied to the annotation of poplar CAZymes in 2006 [17]. Due to its high-quality manual curation and rich functional annotation, CAZy was used as a golden standard dataset to assess automated CAZyme annotation by the CAZymes Analysis Toolkit (CAT) [18] and the dbCAN database [8].

There are also other protein family and function classification databases such as Pfam [19], KOG (eukaryotic orthologous groups) [20], KEGG Orthology (KO) [21], SUPERFAMILY [22], PANTHER [23], Gene Ontology (GO) [24] and many others. Each database has its own strength and focus (e.g. on protein domain or evolution or pathway or structure) and has much redundancy among each other (i.e. one protein family is described in multiple databases). Therefore integration efforts such as InterPro database [25] and CDD database [26] attempted to integrate all these different protein family databases into one framework to remove redundancy. Many of these resources are extremely useful for genome annotation purpose. For example, in the plant genomics community Phytozome [13], Gramene [27] and PLAZA [28] used the above resources to construct and compare protein families across different plants. In addition, ENZYME database [29] created the nomenclature system (i.e. the Enzyme Commission/EC numbers) of all characterized enzymes and associated biochemical reactions. Other databases such as Priam [30], CatFam [31], EFICAz [32] and PlantCyc [33] employed the EC classification system to either define enzyme family models or reconstruct metabolic pathways.

However, unlike CAZy, dbCAN and PlantCAZyme, all the above resources are not specifically designed for CAZymes but rather are general protein family/classification databases. As their mission is to cover all protein families in nature as broadly as possible, they do not have a focus and often miss some families of certain protein class, which is one of the reasons for the need of many specialized databases for individual protein families/classes such as [6, 34–37] (see more at http://www.oxfordjournals.org/nar/database/subcat/3/10). For example, Pfam only covers 142 out of 330 CAZyme families [8]. As a matter of fact, most of these 142 families were initially defined and annotated (from literature curation) by CAZy database and then were included into Pfam as HMMs, which makes Pfam not an ideal resource for CAZyme annotation. In addition, it is well known that one single CAZyme family could contain proteins with different biochemical activities and one biochemical activity could be carried by multiple CAZyme families [4]. For example, the CAZyme GH5 family contains characterized proteins with 20 different EC numbers (manually curated at http://www.cazy.org/GH5.html) and the cellulase (EC 3.2.1.4) activity is found in more than 10 GH families [38]. This makes it impossible to compare dbCAN HMM-based search and EC-based databases (e.g. Priam and CatFam) in terms of CAZyme assignment. Therefore, one cannot evaluate the CAZyme family assignment by comparing to the general protein family/classification databases. Since we aim to assess if we have retrieved all CAZyme homologs using the HMMs built from CAZy annotated proteins, CAZy database is naturally selected as the gold standard dataset to evaluate our performance.

### Accuracy benchmark with *Arabidopsis* and rice data

**Figure 1.**
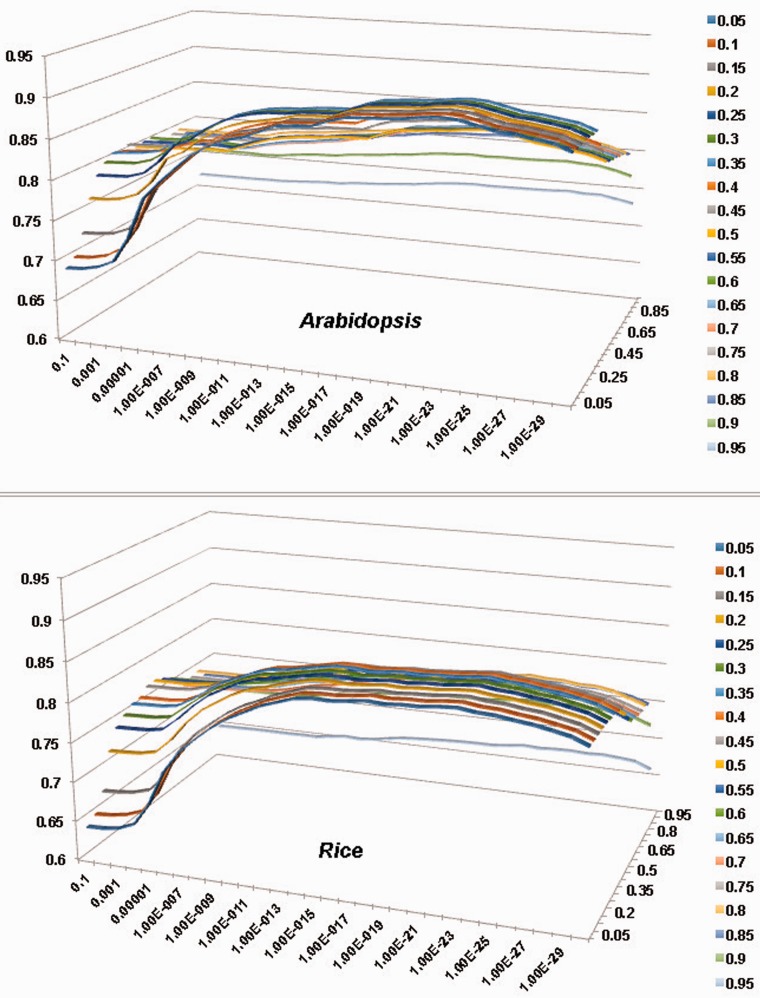
Evaluation of the impact of *E*-value and coverage parameters to the accuracy of pre-computed PlantCAZyme sequence data for *Arabidopsis* and rice; *x*-axis (horizontal): *E-*value, *y*-axis (vertical): F-measure, *Z*-axis: coverage. For both species, *E*-value < 1e–23 and coverage > 0.2 gave the highest F-measure. The detailed calculations are provided in Supplementary Table S1 and S2.

As discussed in our dbCAN article [8], two criteria significantly impact the sensitivity and precision of our automated CAZyme annotation. One is *E*-value and the other is coverage, which is defined to measure the fraction of CAZyme domains covered in the alignment. We have tested the performance of dbCAN-based search on all of the CAZyme families as a whole (denoted as *All*) using different combinations of *E*-values and overage cutoffs. Figure 1 shows the F-measure values of different parameter combinations for the *All* sets of *Arabidopsis* (Figure 1A) and rice (Figure 1B), where *F*-measure = 2 × (Sensitivity × Precision) / (Sensitivity + Precision). We then selected the combination that gave the highest F-measure value and presented them in Tables 2 and 3. The more detailed information about how to calculate Sensitivity and Precision is provided in the Supplementary Tables S1–S12.

Tables 2 and 3 show that the coverage >0.2 and *E*-value < 1*e*-23 combination gave the best F-measure for both *Arabidopsis* (F-measure = 0.91, sensitivity = 0.89 and precision = 0.92) and rice (F-measure = 0.85, sensitivity = 0.84 and precision = 0.85). We have also performed evaluation for the five CAZyme classes separately, which suggests that the best F-measure varies for different CAZyme classes (Tables 2 and 3). Overall the largest two classes GT and GH (81% of CAZyme families) in both plants have higher *F*-measures than the three smaller classes CE, PL and CBM. It also suggests that: (i) to annotate GH proteins, one should use a very relax coverage cutoff or the sensitivity will be low (Supplementary Tables S4 and S9); (ii) to annotate CE families a very stringent *E*-value cutoff and coverage cutoff should be used; otherwise the precision will be very low due to a very high false positive rate (Supplementary Tables S5 and S10). Although it would work best to use different parameter combinations for different CAZyme classes and for different plants, we decided to use coverage > 0.2 and *E-*value < 1**e**-23 as the universal threshold, as this setting agrees in both dicots and monocots and makes the parsing process less complicated and easy to reproduce by others.

**Table 2.** The *E*-value and Coverage cutoffs that lead to the best *F*-measure in *Arabidopsis*

Arabidopsis	# of CAZyme families	*E*-value	Coverage	*F*-measure	Sensitivity	Precision
**All**	98	1.00*E*-23	0.2	0.909236762	0.894071914	0.924924925
**GT**	43	1.00E*-*11	0.25	0.937634409	0.947826087	0.927659574
**GH**	36	1.00*E*-16	0.05	0.974811083	0.969924812	0.979746835
**CE**	5	1.00*E*-29	0.95	0.945741134	0.917647059	0.975609756
**PL**	2	1.00*E*-30	0.25	0.970588235	0.970588235	0.970588235
**CBM**	10	1.00*E*-12	0.75	0.79613773	0.821428571	0.772357724

**Table 3.** The *E*-value and coverage cutoffs that lead to the best *F*-measure in Rice

Rice	# of CAZyme families	*E*-value	Coverage	*F*-measure	Sensitivity	Precision
**All**	97	1.00*E*-23	0.2	0.845169681	0.840619308	0.849769585
**GT**	44	1.00*E*-10	0.35	0.906381793	0.908931699	0.903846154
**GH**	35	1.00*E*-13	0.1	0.92415331	0.91745283	0.930952381
**CE**	5	1.00*E*-28	0.95	0.913545252	0.905660377	0.921568627
**PL**	2	1.00*E*-30	0.7	0.827586207	0.75	0.923076923
**CBM**	9	1.00*E*-16	0.45	0.716031632	0.857142857	0.614814815

### Annotation data

**Figure 2.**
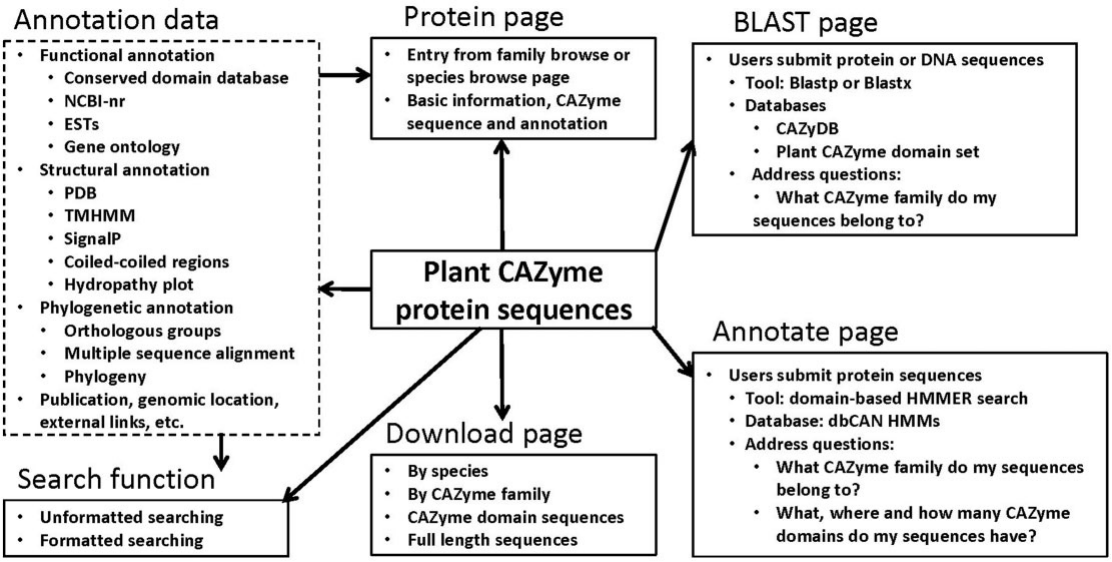
A schematic architecture of the PlantCAZyme database

We have further generated extensive bioinformatics annotation data for the plant CAZyme sequences by running various bioinformatics tools against different databases. As shown in Figure 2, these data include functional annotation (conserved functional domains, Gene Ontology annotation, top matches in the non-redundant protein database [NCBI-nr] and expressed sequence tag (EST) database), structural annotation [top matches in the Protein Data Bank (PDB), predicted transmembrane domains, signal peptides, coiled regions, hydropathy plot], phylogenetic annotation (orthologous groups of the CAZyme domains, multiple sequence alignment, phylogenetic tree) and miscellaneous data (nucleotide coding sequences, CAZyme signature domain sequences, genomic location, external links, publications, etc.).

## Utility and Discussion

### Implementation and user interface

All the data were integrated and presented through a web interface powered by MySQL+PHP+JavaScript. As shown in Figure 2, the *protein centric display page* is used to present the sequence and annotation of each CAZyme protein. The website has a *download page* that allows users to download CAZyme sequences of a particular species or a particular CAZyme family. Both the CAZyme signature domain sequences and the full-length sequences are available for any species or any family.

A *BLAST page* and a HMMER (annotate) page were included to allow users to submit their own sequences for annotation, which are very useful to annotate sequences that are not included in our database. For BLAST search, users can submit both protein and nucleotide sequences and the databases for BLAST search can be chosen from: (i) the CAZy database that contains full-length GenBank protein sequences annotated in the CAZy database, (ii) the plant CAZyme domain sequences (not the full length) that are compiled in our PlantCAZyme database containing the CAZyme signature domains identified by dbCAN search. The results are returned as a webpage with a tabular output of the BLAST program.

For *HMMER page*, users must submit protein sequences as query and the database is the dbCAN’s HMMs. Since HMMs are built for each CAZyme family to represent the signature domain, this type of search is a better way than BLAST search to annotate new protein sequences with the modular CAZyme domain architecture.

In addition to sequence search, the *keyword search* function was also implemented. The top-right corner of each webpage has a search box, where users can search the database with a keyword. There are two options for keyword search: unformatted searching and formatted searching. For unformatted searching you enter a query with no formatting. This will run the query only against the following fields: (i) ID, e.g. AT2G46570.1, (ii) Family, e.g. CBM10, (iii) Species, e.g. *A.* *thaliana* and (iv) Domain, e.g. Cellulose_synt. Formatted searching allows users to be more specific and search through more fields. Formatted searches are done by indicating formatting with the use of brackets []. For example, if users want to search for the species *A.* *thaliana*, they can search ‘*Arabidopsis thaliana*[Species]’, which will bring up anything with a species containing ‘Arabidopsis’ or ‘thaliana’. Users can write more than one specifier in a query. So if users only wanted the AA1 family, they could write the query as ‘Arabidopsis[Species] thaliana[Species] AA1[Family]’. These specifiers are all strung together in an AND fashion, so a result will only appear if it matches all of the criteria users have given. Currently the keyword search only allows exact match and does not allow partial match and wildcard, which will be considered in the future.

A help page is designed to provide all necessary information for browsing, querying, downloading and searching the website and the database.

## Use cases

If users want to retrieve all CAZyme proteins of *A. thaliana*, there will be three options. (i) Users can go to the download page, browse by species and locate the species to download the FASTA format sequences of full-length proteins or just the CAZyme domains. (ii) They can also go to the homepage, browse by species, click on the species and link to the family browse page of *A. thaliana*. There they can view which CAZyme families are in *A. thaliana* and how many genes are in each family, as well as a clickable genomic location plot. This *Arabidopsis thaliana* browse page also has a link to the complete HMMER output, where hits that did not pass our filters (coverage > 0.3 and *E*-value < 1*e*-5) can also be retrieved. Clicking on each family will present a new page with the list of proteins of that family, and further clicking on the ID will open the protein browse page. (iii) The last way is to perform a keyword search in the following format: *(Arabidopsis thaliana)[species]* or *Arabidopsis[Species] thaliana[Species]*, which will return a table with all the *Arabidopsis thaliana* CAZyme IDs.

Similarly, if users want to retrieve CAZyme proteins of a specific family, say GT8, they will have the three options too: (i) download all GT8 proteins at the download page, (ii) browse by family at the homepage and (iii) use the keyword search function: *GT8[family]*.

If users have a dataset (e.g. a newly sequenced genome) to be annotated for CAZymes, they can upload the FASTA sequences to our computing server through the BLAST page or the annotate (HMMER) page. The job will be run and the result will be returned with the CAZyme match information. If a huge dataset (>5000 sequences) needs to be processed, we recommend that users download the BLAST databases (CAZyDB or PlantCAZyme) or the HMM database (dbCAN) at our download page and run the searches on their local computers.

### Future work

We plan to update the database at least once a year. We plan to include more species in the future, particularly selected plants and algae that do not have completed genomes. We will use transcriptomes of species such as ferns, liverworts, charophytic green algae (CGA), basal angiosperms, as they are important for the evolutionary study of CAZymes in plants and algae. The automatic collection of CAZyme sequences will also be further improved, e.g. by considering applying different parsing thresholds for different plant clades and by supplementing the HMMER search with BLAST search. We will also develop new web applications to display duplicated genes and orthologous genes of CAZymes on the chromosomes to allow comparative and evolutionary study of CAZymes.

*PlantCAZyme* is the first web resource dedicated to provide pre-computed CAZyme sequence and annotation data for all sequenced plants and algae. We expect it will be a highly useful tool to the plant cell wall and bioenergy research communities.
